# Case Report: Detection and characterization of avian influenza virus H9N2 in a broiler farm in Mozambique, 2025

**DOI:** 10.3389/fvets.2026.1765651

**Published:** 2026-03-24

**Authors:** Iolanda Vieira Anahory Monjane, Virgínia Nhabomba, Esmeralda Tamele, Hernâni Djedje, Afonso Sussuro, Teresa Marlene Van-Dúnem, Ilda Mandlate, Dercília M. Arone, Zacarias E. Massicame, Job Massingue, Almiro R. Tivane, Jorfélia J. Chilaúle, Daisy Zacarias, Dércio Chaúque, Domingos Mulungo, Charles Euloge Lamien, Irene Kasindi Meki, William G. Dundon

**Affiliations:** 1 Central Veterinary Laboratory, Directorate of Animal Science, Agrarian Research Institute of Mozambique, Maputo, Mozambique; 2 National Directorate of Health and Biosafety, Ministry of Agriculture Ambient and Fishery, Maputo, Mozambique; 3 Provincial Directorate of Agriculture and Fisheries, Maputo, Mozambique; 4 Respiratory Virus Surveillance System, Instituto Nacional de Saúde, Maputo, Mozambique; 5 Virology Laboratory, Instituto Nacional de Saúde, Maputo, Mozambique; 6 Animal Production and Health Laboratory, Joint FAO/IAEA Centre of Nuclear Techniques in Food and Agriculture, Department of Nuclear Sciences and Applications, International Atomic Energy Agency, Vienna, Austria

**Keywords:** avian influenza, broiler, G5.5, genome, H9N2, Mozambique

## Abstract

In August 2025, Mozambique's Central Veterinary Laboratory, part of the Agricultural Research Institute, received broiler chicken carcasses for investigation after reports of elevated flock mortality. Pooled samples from the carcasses (i.e. trachea and lung) tested positive for influenza A. Subsequent subtyping ruled out hemagglutinin subtypes H5 and H7. Further analysis including tests for Newcastle disease virus, infectious bronchitis virus, infectious laryngotracheitis, and avian influenza subtype H9 confirmed the presence of H9. Whole-genome sequencing using Nanopore technology identified the virus as H9N2. Phylogenetic analysis of the hemagglutinin gene classified the virus within lineage G5.5, showing closer genetic similarity to strains circulating in the Middle East (e.g., Saudi Arabia, Jordan, and Israel) than to those in East or West Africa. This report is the first documented detection of H9N2 avian influenza in Mozambique, with significant implications for regional food security and disease management.

## 1 Introduction

Low pathogenic avian influenza viruses of the H9N2 subtype have emerged as a significant threat to poultry health over the past few decades. While infections are often mild, the virus can cause respiratory distress, reduced egg production and feed intake, and, through secondary infections, more severe outcomes, including mortality. Beyond its impact on poultry, H9N2 also poses an increasing public health risk due to its zoonotic potential and ability to infect humans (1).

In 2024, Fusaro et al. (2) introduced a standardized classification system for H9N2 viruses, superseding earlier systems. Current classification defines three primary lineages, B, G, and Y, along with first-, second-, and third-order clades. Among these, the Y lineage exhibits the broadest global distribution, with viruses detected across Africa, the Americas, the Asia-Pacific region, Oceania, and Europe. The G lineage is predominantly reported in the Middle East and South Asia, though it has also been identified in Africa and Europe. In contrast, the B lineage, which has the most limited distribution, is confined to the Asia-Pacific region and European Russia (2).

H9N2 was first detected on the African continent in South Africa in 1995, followed by reports in Egypt and Libya in 2006, Tunisia in 2010, and Morocco in 2016 (1, 3). In West Africa, the virus emerged in Burkina Faso in 2017 and has since been identified and characterized in Senegal, Mali, Ghana, Nigeria, Benin, Togo (4–8), and most recently in Mauritania (9). In East Africa, H9N2 outbreaks have been reported in Uganda, Kenya, and Madagascar (10, 11). H9N2 in South Africa has been associated with mild, localized outbreaks, primarily in ostriches and occasionally in other bird species, with no recent large-scale or high-impact events (12).

The poultry sector is a vital component of Mozambique's agricultural economy, contributing to food security, employment, and rural livelihoods. The country produces an estimated 135,000 tons of chicken meat annually according to the Ministry of Agriculture and Rural Development (www.agriculura.gov.mz), which corresponds to roughly 135–150 million birds. In 2024, the country also produced about 360 million eggs, reflecting steady growth in the sector. Small-scale and backyard farming dominate, accounting for over 80% of national poultry production, while commercial farms supply urban markets. Commercial poultry farming is growing, driven by urban demand, but faces challenges from infectious disease like Newcastle Disease (ND), Infectious Bronchitis Virus (IBV) and avian influenza (AI). In 2023, an outbreak of a novel H7N6 highly pathogenic avian influenza was reported 500 km northeast of the capital Maputo (13). The outbreak was contained through movement restrictions, culling of infected birds, and the destruction of contaminated feed and eggs.

This case report provides details of the first H9N2 AI outbreak reported in Mozambique and the subsequent molecular characterization of the causative virus.

## 2 Case description

On July 4th, 2025, a commercial chicken farm (Farm 1) located in Mukatine, Matola district in Maputo Province (GPS: 25°44'10.3"S; 32°32'19.5"E) acquired 1,500-day-old chicks (vaccinated against NDV) for fattening and slaughter from two local suppliers. In the following 3 weeks the farmer observed sick birds showing the following signs: lethargy, respiratory distress, yellowish diarrhea, coughing, nasal and ocular discharge.

The poultry house consisted of one building divided into four compartments. The sick birds were separated by compartments from the apparently healthy birds. Birds began to die at a rate of 15–20 per day reaching a total of approximately 350 (23%). To avoid further losses, the farmer sold 500 apparently healthy birds at 37 days of age to local rotisserie chicken vendors.

## 3 Diagnostic and outbreak assessment

### 3.1 Laboratory diagnosis

On the 15th of August 2025, the Central Veterinary Laboratory of the Agricultural Research Institute of Mozambique received 10 randomly selected chicken carcasses from the farm for disease investigation. External observations revealed facial oedema, cyanosis of the comb and wattles and nasal discharge while internal observations identified congestion and inflammation of the lungs. Total RNA was extracted from pooled samples (i.e. trachea and lung) using the QIAamp Viral RNA mini kit (Qiagen, Germany). The RNA was tested for the presence of influenza Type A, by real-time RT-PCR using standard protocols (14). Purified RNA from two positive samples was sent to the Animal Production and Health Laboratory, Joint FAO/IAEA, Seibersdorf, Austria, for further analysis. On arrival in Austria, the RNA was tested using a multiplex assay that identifies influenza Type A, Newcastle disease virus (NDV), infectious bronchitis virus (IBV), and infectious laryngotracheitis virus (ILTV) (15). The RNA was also tested using a protocol which identifies H9 subtype viruses (16).

### 3.2 Outbreak assessment

A multidisciplinary and multisectoral team from the Agrarian Research Institute of Mozambique-Directorate of Animal Science, National Institute of Health, Maputo Provincial Agriculture and Fisheries Services, National Directorate of Plant and Animal Health and Biosecurity, and the National Directorate of Environment and Climate Change—Waste Disposal Department was dispatched on the 16 August 2025 to the affected farm. To determine the extent and magnitude of the outbreak, surveillance activities were conducted around the affected farm. Five nearby poultry farms were visited. In addition, a trace-back investigation was carried out through telephone interviews with all farmers who had purchased chicks from the same supplier companies.

### 3.3 . Control measures implemented

Under the supervision of a technician from the Directorate of Environment, culling and destruction of all birds (both sick and healthy), feed, and poultry litter and disinfection of equipment from the affected farm was undertaken.

### 3.4 Whole genome sequencing

The purified RNA from one of the positive samples obtained from Farm 1 and renamed A/chicken/Mozambique/429/2025 was amplified according to the protocol of Zhou et al. (17) with modifications. Briefly, 5 μl of total RNA was added to a reaction mixture consisting of 0.8 μl of primer MTBUni-12 (5′ GCGTGATCAGCAAAAGCAGG 3′) (5 μM), 1.2 μl of primer MTBUni-12.4 (5′ GCGTGATCAGCGAAAGCAGG 3′) (5 μM), 1 μl of primer of primer MTBUni-13 (5′ ACGCGTGATCAGTAGAAACAAGG3′), 25 μl of 2X reaction mix (SuperScript^TM^ III, Invitrogen), 4 μl of MgS0_4_ (10 mM), 1 μl of Superscript III reverse transcriptase-Platinum *Taq* High Fidelity Enzyme (Invitrogen) and nuclease free water to a final volume of 50 μl. The thermocycling conditions were reverse transcription at 55 °C for 60 min, denaturation at 94 °C for 2 min, followed by five cycles of 94 °C for 30 s, 45 °C for 30 s, and 68 °C for 4 min and a further 31 cycles of 94 °C for 30 s, 57 °C for 30 s, and 68 °C for 4 min with a final elongation at 68 °C for 5 min.

The PCR amplicons were purified using 0.5X AMPure XP beads (Beckman Coulter, CA, USA) prior to library preparation and sequencing on an Oxford Nanopore MinION Mk1D (Oxford Nanopore Technologies, UK). Libraries were prepared using the Ligation Sequencing Kit V14 (cat# SQK-LSK114) following Oxford Nanopore Technologies standard protocols. Briefly, 200 ng (~200 fmol) of the purified amplicons underwent end repair using the NEBNext Ultra II End repair kit (New England Biolabs, MA, USA) followed by adapter ligation. The resulting libraries were purified with AMPure XP beads and 50 ng (~50 fmol) was loaded onto an R10.4.1 MinION flow cell for sequencing. Data acquisition and high-accuracy basecalling were performed using MinKNOW v25.05.12 with integrated Dorado v7.9.8 (Oxford Nanopore Technologies, UK).

The generated fastq files from sequencing were processed using an in-house bash pipeline. The workflow applies porechop to clean the reads and adaptor trimming, chopper (v7) for filtering low-quality and short reads, and NanoPlot for quality assessment of the clean reads. The clean reads were first classified using Centrifuge (v.1.0.4), using a minimum hit-length of 35, and the top hits were extracted and visualized in a pie chart. The clean reads were aligned to H9N2 reference sequences for all segments using Minimap2 (v2.26) and visualized using Integrative Genome Viewer (IGV) (https://igv.org). SAMtools (v1.18) generated Mpileup files, BCFtools (v1.18) performed variant calling and consensus generation, and the consensus sequences were extracted using vcfutils.pl (VCFtools v0.1.16) and seqtk (v1.3). The genome coverage was assessed using Qualimap (v2.3).

Phylogenetic analysis of the hemagglutinin (HA) sequence (1,485 bp) of A/chicken/Mozambique/429/2025 was performed using the maximum-likelihood (ML) method available in MEGA 11 (18) employing the Tamura-Nei model of nucleotide substitution with uniform rates among sites and 1000 bootstrap replications.

## 4 Results

### 4.1 Outbreak assessment

Of the five farms visited by the investigation team, one farm reported a 50% bird mortality rate. The owner of this farm had recently purchased chicks from a local seller. A total of 75 farmers from the provinces of Maputo and Gaza who had acquired chicks from 3 different sources in Maputo on 4 July were interviewed by telephone. Among these, two farms reported clinical signs identical to those observed in the affected farms, including diarrhea, tearing, respiratory distress, and poor growth.

### 4.2 Laboratory diagnosis

The analysis of the samples obtained from Farm 1 and those collected from three other farms as part of the outbreak assessment were positive for influenza Type A and H9 subtype and negative for H5, H7, NDV, IBV, and ILTV.

### 4.3 Whole genome sequencing and analysis

A complete coding sequence was obtained for all eight segments of A/chicken/Mozambique/429/2025, with mean coverage ranging from 417x to 61,848x. Segment-specific mean coverage were as follows: polymerase basic protein 2 (PB2, 895x), polymerase basic protein 1 (PB1, 554x), polymerase basic protein (PA, 417x), hemagglutinin (HA, 2,426x), nucleoprotein (NP, 7,950x), neuraminidase (NA, 1,170x), matrix protein (M, 46,239x), and non-structural protein (NS, 61,848x). A BLASTn analysis of the nucleotide sequence from each of the genome segments, within the GISAID database (www.gisaid.org), revealed that all of the segments, except for the HA, had the highest nucleotide identity (98.60 to 99.80 %) to a G5.5 lineage H9N2 virus identified in Saudia Arabia in 2022 (A/Chicken/SA/S5/2022) (Table 1). The HA of A/chicken/Mozambique/429/2025 had a higher nucleotide identity with the HA from a virus from Jordan in 2024 (A/chicken/Jordan/24-10/2024). Only the HA sequence is available for A/chicken/Jordan/24-10/2024; it is presumed that if the other segments of A/chicken/Jordan/24-10/2024 were available that this virus would be the closest relative to A/chicken/Mozambique/429/2025. Nevertheless, it can be concluded that A/chicken/Mozambique/429/2.025 is not a reassortant.

**Table 1:** Comparison of the genome segments of A/chicken/Mozambique/429/2025 with their nearest relatives.

**Segment**	**A/chicken/Mozambique/426/2025**	**GISAID#**	**%**
PB2	A/Chicken/SA/S5/2022	EPI4203184	98.72
PB1	A/Chicken/SA/S5/2022	EPI4203185	98.99
PA	A/Chicken/SA/S5/2022	EPI4203186	99.44
HA	A/chicken/Jordan/24-10/2024 A/Chicken/SA/S5/2022	EPI3549597 EPI4203187	99.12 98.46
NP	A/Chicken/SA/S5/2022	EPI4203197	99.06
NA	A/Chicken/SA/S5/2022	EPI4203171	98.60
M	A/Chicken/SA/S5/2022	EPI4203199	99.80
NEP	A/Chicken/SA/S5/2022	EPI4203201	98.69

**Figure 1:**
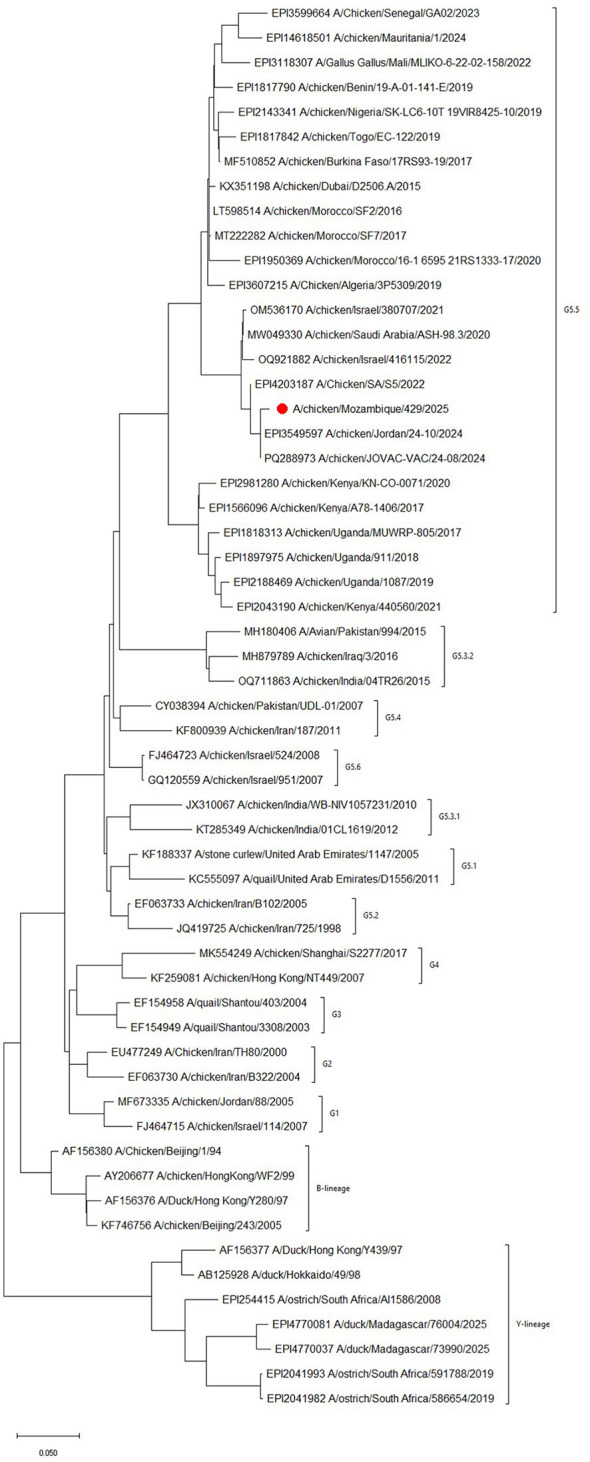
Maximum-likelihood phylogenetic tree of the HA gene (1485 bp). A/chicken/Mozambique/429/2025 from this study is highlighted with a red dot. Lineages are shown.

The phylogenetic tree obtained using 1485 bp of the HA sequence of A/chicken/Mozambique/429/2025 (Figure 1) clearly showed that the sequences clustered with G5.5 lineage H9N2 virus identified in Saudia Arabia, Israel and Jordan between 2020 and 2024.

The genome of the A/chicken/Mozambique/426/2025 virus was analyzed to identify molecular markers associated with enhanced virulence and host tropism. The HA cleavage site motif, RSSRG/LF, is consistent with motifs observed in H9N2 viruses previously identified in African countries. Notably, the virus exhibited HA substitutions at positions 191 (H) and 234 (L), which are associated with a shift in receptor binding preference from avian α-2,3 sialic acid receptors to human α-2,6 sialic acid receptors, suggesting potential for human infection (19). Additionally, a neuraminidase (NA) substitution, R403W, was detected, a mutation known to facilitate adaptation to mammalian hosts (20). In the polymerase basic protein 2 (PB2), residues 185I and 588V were identified. These are linked to increased viral pathogenicity in mice and enhanced polymerase activity and replication in mammals, respectively (21, 22). The PB1-F2 protein was truncated to 52 amino acids, compared to its typical length of 90 amino acids, removing residues implicated in modulating the host immune response and potentially influencing pathogenesis (23). The polymerase acidic protein (PA) contained an N291S substitution, which has been associated with heightened pathogenicity in mice (21). Finally, the matrix protein 2 (M2) exhibited the S31N mutation, conferring resistance to amantadine (24).

## 5 Discussion

This study presents the first documented case of genotype G5.5 H9N2 AI in Mozambique. To date, reports of H9N2 in the region have been limited to ostriches and wild birds in South Africa (12) and ducks in Madagascar (GISAID) but these viruses belong to lineage Y as shown in the phylogenetic analysis from this study and, as such, are genetically distinct from A/chicken/Mozambique/429/2025.

The closest genetic relatives of the A/chicken/Mozambique/429/2025 strain are G5.5 lineage viruses identified in the Middle East, which is unusual given the lack of evident epidemiological links between that region and Mozambique. There are no official records of poultry or poultry product imports from the Middle East to Mozambique, raising questions about the origin of this introduction. Although, while wild bird migration could potentially explain the arrival of H9N2 in Mozambique (12, 25–27), it is also possible that the virus entered Mozambique from a neighboring country but this is difficult to ascertain based on the limited data available on AI in countries bordering Mozambique such as Tanzania, Malawi, Zambia, and Zimbabwe. Identification and reporting of similar outbreaks in the region would significantly improve our understanding of the movement and impact of AI viruses of all subtypes.

How the virus entered the farm is also unclear. A biosecurity assessment revealed several critical weaknesses that significantly increase the risk of pathogen introduction and spread. The farm was located adjacent to a secondary road and lacked a perimeter fence or physical barriers, allowing unrestricted access for people, vehicles, and stray animals. This absence of controlled entry heightens the risk of introducing infectious agents via footwear, equipment, or animal carriers. While footbaths were present, they did not contain disinfectant, making them ineffective. Furthermore, there were no designated disinfection points for vehicles, equipment, or visitors, which undermines sanitary control measures. The processing room, storeroom, changing rooms, and incubator were situated in close proximity to the poultry houses without physical separation. This layout promotes cross-contamination between clean and potentially contaminated areas, compromising the entire production chain. The farm did not follow the “All-in, All-out” system, which is crucial for breaking infection cycles, enabling thorough cleaning, and ensuring adequate sanitary rest periods. External individuals, including buyers, frequently entered the henhouses to select birds, directly exposing the flock to biological risks. Additionally, there was a lack of accessible records for mortality, production metrics, treatments, and other essential data required for sanitary control and disease monitoring. This absence of documentation impedes early outbreak detection.

The high mortality (23%) observed is unusual for a flock infected with H9N2. Nevertheless, it is known that viral co-infections, specifically infectious bronchitis virus (28, 29) and secondary bacterial infections (e.g., *Mycoplasma gallisepticum, Staphylococcus aureus, Escherichia coli, Ornithobacterium rhinotracheale)* can occur following H9N2 infection which can result in increased mortalities (30, 31). The samples were tested for IBV and were negative. Unfortunately, the samples were not screened for secondary pathogens.

This is only the second report of AI in Mozambique, the first being a single report on an H7N6 outbreak in 2023 (13). Given the recorded ability of H9N2 to reassort with other AI viruses including H5 and H7 subtypes, there is a potential risk associated with the circulation of H9N2 in Mozambique. Although H5 subtype viruses have not been reported in Mozambique to date, H5N1 and H5N8 subtype viruses have been identified in southern African countries like Botswana, Lesotho, Namibia and South Africa both in domestic and wild birds (12, 32–35).

The findings of this study carry significant implications for Mozambique's poultry industry, regional food security, and public health, both within the country and in neighboring nations. As the poultry sector plays a crucial role in Mozambique's food security, any threat to its productivity demands urgent attention from relevant authorities. Furthermore, the zoonotic potential of circulating H9N2 viruses must not be overlooked. To address these challenges, national authorities in Mozambique should prioritize the following actions; (1) strengthening surveillance programs and expanding diagnostic laboratory capacity, including virus isolation and characterization,; (2) supporting the implementation of disease mitigation and management strategies, such as vaccination programs where feasible; (3) promoting the dissemination of training on prevention and biosecurity measures among poultry associations, workers, and smallholder farmers to safeguard their flocks.

## Data Availability

The datasets presented in this study can be found in online repositories. The names of the repository/repositories and accession number(s) can be found below: https://www.ncbi.nlm.nih.gov/genbank, PX492092-PX492099.
